# Effects of suspended micro- and nanoscale particles on zooplankton functional diversity of drainage system reservoirs at an open-pit mine

**DOI:** 10.1038/s41598-019-52542-6

**Published:** 2019-11-06

**Authors:** Anna Maria Goździejewska, Monika Gwoździk, Sławomir Kulesza, Mirosław Bramowicz, Jacek Koszałka

**Affiliations:** 1 Faculty of Environmental Science, University of Warmia and Mazury in Olsztyn, Oczapowskiego 5, 10-719 Olsztyn, Poland; 2 Faculty of Production Engineering and Materials Technology, Czestochowa University of Technology, Armii Krajowej 19, 42-200 Częstochowa, Poland; 3 Faculty of Mathematics and Computer Science, University of Warmia and Mazury in Olsztyn, Słoneczna 54, 10-710 Olsztyn, Poland; 4 Faculty of Technical Science, University of Warmia and Mazury in Olsztyn, Oczapowskiego 11, 10-719 Olsztyn, Poland

**Keywords:** Freshwater ecology, Limnology

## Abstract

Water from mining drainage is turbid because of suspensions. We tested the hypothesis that the chemical composition as well as shape and size of particles in suspensions of natural origin affect the density and functional diversity of zooplankton. The suspensions were analyzed with atomic force microscopy (AFM), energy dispersive X-ray spectroscopy (EDS), scanning electron microscopy (SEM), and optical microscopy. Elements found in the beidellite clays were also identified in the mineral structure of the particles. As the size of the microparticles decreased, the weight proportions of phosphorus, sulfur, and chlorine increased in the suspensions. These conditions facilitated the biomass growth of large and small microphages and raptorials. As the size of the nanoparticles decreased, the shares of silicon, aluminum, iron, and magnesium increased. These conditions inhibited raptorials the most. Ecosystem functionality was the highest with intermediate suspension parameters, which were at the lower range of the microphase and the upper range of the nanophase. The functional traits of zooplankton demonstrate their potential for use as sensitive indicators of disruptions in aquatic ecosystems that are linked with the presence of suspensions, and they facilitate gaining an understanding of the causes and scales of the impact of suspensions.

## Introduction

Processes associated with open-pit mining has serious impacts on natural environment, including aquatic ecosystems. Drainage water is found turbid and carry high loads of suspensions. To reduce pollution and sustain required water quality, drainage water is directed to settling reservoirs prior to discharge into the rivers. Apart from this primary function, however, settling reservoirs are also used as fisheries and places for various socio-recreational activities^1^.

Suspensions are natural components of aquatic ecosystems, usually made of solid particles with diameters less than 60 µm^2^. They participate in many biological, physical, and chemical processes^3–6^ depending on their origin, concentration and type. Suspensions rich in organic matter are valuable food sources for microorganisms and subsequent species in the consumer chain. In turn, inorganic particles are necessary in the cycling of trace elements^7–9^ and act as substrates for the development of algae and protozoans^10^. Unfortunately, at higher concentrations suspensions may disrupt trophic processes affecting, for example: primary production due to limited light penetration, feeding effectiveness, development and abundance of filtering organisms etc^11–14^. High cloudiness of water may also limit the foraging success of fishes that locate preys visually^15,16^.

Depending on actual concentration, chemical composition, and particle size, inorganic wastes in water created in large quantities during extraction of minerals seriously affect aquatic organisms^6^. This explains why biological significance of nanosized particulate suspensions is gaining increasing attention recently. Although nanoparticles are known to be intrinsic components of the natural environment, we observe sharply rising amount of nanoparticles released from industrial processes^17^. Nanoparticles are found to be the basal building blocks of complex systems such as, *inter alia*: sorption sites, process triggers in catalysis, assembled complexes, functional structures, fillers, etc., and their properties are known substantially enhanced in comparison with their solid counterparts^18,19^. With decreasing particle size, the surface-to-volume ratio of the particles increase in reciprocal proportion leading to qualitative change in their properties. The need for supramolecular particles with extraordinary properties has stimulated large scientific interest in nanoparticles. In order to gain an insight into such small objects, atomic force microscopy (AFM) appears to be an unique tool capable of characterizing both spatial geometry and mechanical properties on a nanoscale. To date, there is limited number of papers that report characterization of surface irregularities of suspension particles by means of surface-to-volume ratio, which is extremely important parameter in determining various phenomena influencing the environment. Surface roughness, geometry, and particle concentration appear to be key factors in this respect^20,21^.

Recently, many authors have reported on the impact the particles associated with the production of nanomaterials are having on aquatic organisms^22–25^. Mineral macro- and microparticles are caught directly from the environment with food and can mechanically disrupt processes of feeding and digestion^26,27^. On the contrary, nanoparticles are introduced via endocytic pathways. Their reactivity stems not only from the size of the active surface, but also from a substantially larger scale of impact^28,29^. Studies on the influence of nanoparticles upon planktonic invertebrates were carried out with toxicology tests and referred mostly to Cladocera species^22,25,30,31^. Unfortunately, there are no similar studies on zooplankton in natural environment.

The suitability of zooplankton in evaluating environmental disruptions stems from its versatile bioindication features. These bioindicative traits include: wide distribution in different aquatic ecosystems, significance in nutrient and energy cycling, short life cycles, adaptation to environmental changes, and sensitivity to sudden environmental disturbances (pollution, water flow and level, thermal conditions, salinity, acidification, etc.)^32–37^. Alternative approach makes use of functional traits of the zooplankton (behavioral and morphological diversities related to feeding strategy). For example, it was demonstrated that the diversity and seasonal variation in domination of microphagous and predatory zooplankton species can be a measure of the resilience of aquatic ecosystems^32,35^. This means that the environment creates an extensive trophic network and is rich in species and habitats. This contrasts with a state of trophic asymmetry (the continual domination of a trophic group), which is an indicator of the reaction of the environment to anthropogenic stress^38,39^. An ecosystem that is exposed to radical disruption factors comprises species that are functionally similar; therefore, it loses its functionality compared to environments in which the species are less functionally similar^40^. Thus, the zooplankton structure reflects on-going phenomena and processes in aquatic basins^32,41–43^.

The distribution and structure of planktonic communities is explained by the intermediate disturbance hypothesis proposed by Connell^44^. When disturbances occur too often (e.g. river water flow), populations characterized by lower growth rates (e.g. Cladocera) soon are becoming extinct. On the other hand, if the disturbances are rare, the system reaches a competitive equilibrium, in which the species with low competitive ability are eliminated (e.g. eutrophic habitats of the lakes). Between these two extreme cases is an intermediate level of disturbance that maximizes species diversity^34^.

As mentioned previously, functional zooplankton traits can be an effective tool in assessing biological potential of aquatic ecosystems^45^. Goździejewska *et al*.^1^ were the first who studied the relation between physico-chemical parameters of post-mining waters and the structure of zooplankton assemblages in settling reservoirs. They confirmed the effect of good feeding conditions on zooplankton abundance similar to that in natural aquatic ecosystems except for the concentration of suspensions. Obtained results have led us to the question, how zooplankton communities deal with natural particles suspended in waters from settling reservoirs in open-pit mines, which was undertaken in the current work. According to that, performed research aimed at, as follows: (1) determination of key properties of suspensions that are responsible for shaping zooplankton species and trophic structure, (2) assessment of the nature of the above relation (direct vs. indirect), (3) derivation of the functionality of ecosystems and their resistance to disruptions caused by suspensions (based on zooplankton functional diversity), (4) examination of the applicability of the Connell’s “intermediate disturbance hypothesis”^44^ in investigations of settling reservoirs.

Understanding the factors responsible for the diversity of functional feeding guilds of zooplankton would allow to forecasting the dynamics of aquatic ecosystems. This knowledge is extremely significant in order to minimize the environmental aftereffects of open-pit mining in Europe.

## Results

### Hydrochemical properties of water

The highest average concentrations of the suspensions were noted in samples KA1 and KU (9.5 mg L^−1^ and 8.5 mg L^−1^, respectively), though this parameter did not vary significantly among the reservoirs under study (Table 1). The highest inorganic fraction was found in samples KA3 and CH1 equal to 67 and 60%, respectively, while in the remaining samples the dominance of organic particles was observed (52–62%). Note also a strong correspondence between concentration of suspensions and water turbidity (Supplementary Table S1). The lowest turbidity was found in WI and PN (5 NTU), whereas the highest in KU (36 NTU) and KA1 (29 NTU). The differences in these quantities proved their statistical significance (*P* ≤ 0.05; Table 1). In addition, increasing turbidity was associated with decreasing SDT values (Supplementary Table S1). The largest SDT was found in WI (1.2–1.7 m), while the lowest in KU (0.4–0.6 m). Finally, there was a dependence between the concentration of inorganic suspensions and the water color (Supplementary Table S1). The highest water color was noted in KU (25–47 Hazen), while the lowest in WI and PN (7–10 Hazen). Differences in the mean values of the transparency and color parameters passed the test of statistical significance (*P* ≤ 0.05; Table 1).

**Table 1:** Location, morphometric, and water quality parameters of the studied reservoirs (mean ± SD).

Reservoir	Water supply	Geographical coordinates	Area (ha)	Depth (m)	Age (yr)	Temp (C)	pH	DO (mg L^−1^)	SDT (m)	Color HAZEN	Turbidity NTU	Tot susp (mg L^−1^)	In susp (mg L^1-^)	Org susp (mg L^1-^)	Chl *a* (µg L^−1^)	TP (mg L^−1^)	TN (mg L^−1^)
CH1	OM-S	51º15′58.9″N 19º06′24.3″E	8.1	2.6	13	20.0 ± 1.8	7.32 ± 0.22	8.33 ± 1.89	0.80^ab^ ± 0.28	18.50^ab^ ± 11.7	17.25^ab^ ± 13.00	6.56 ± 6.51	3.26 ± 2.19	3.30 ± 4.43	4.34^ab^ ± 2.34	0.149 ± 0.04	0.390 ± 0.14
CH2	OM-S	51º15′58.9″N 19º06′24.3″E	7.9	2.6	13	19.6 ± 1.9	7.32 ± 0.31	7.88 ± 0.60	0.80^ab^ ± 0.29	16.25^ab^ ± 6.18	14.00^ab^ ± 7.07	7.21 ± 3.49	2.55 ± 1.04	4.66 ± 3.17	4.96^ab^ ± 2.68	0.166 ± 0.03	0.390 ± 0.05
KA1	OM-B	51º15′18.9″N 19º12′15.4″E	7.1	2.7	20	19.8 ± 2.8	7.50 ± 0.08	8.28 ± 0.78	0.65^b^ ± 0.02	24.00^ab^ ± 5.59	27.50^b^ ± 2.38	9.48 ± 5.96	2.82 ± 1.19	6.66 ± 5.84	16.76^b^ ± 15.56	0.175 ± 0.02	0.378 ± 0.14
KA2	OM-B	51º15′18.9″N 19º12′15.4″E	7.1	2.7	20	19.1 ± 2.5	7.48 ± 0.03	8.16 ± 0.39	0.68^b^ ± 0.06	24.5^ab^ ± 8.34	24.00^ab^ ± 3.26	7.61 ± 3.52	3.28 ± 2.28	4.33 ± 1.71	10.08^b^ ± 4.34	0.155 ± 0.02	0.287 ± 0.09
KA3	OM-B	51º15′18.9″N 19º12′15.4″E	7.1	2.7	20	19.7 ± 2.7	7.49 ± 0.22	7.44 ± 1.14	0.68^ab^ ± 0.17	19.5^ab^ ± 5.06	13.25^ab^ ± 4.78	7.04 ± 10.23	1.57 ± 0.68	5.47 ± 10.18	3.78^ab^ ± 3.03	0.138 ± 0.07	0.368 ± 0.16
KU	OM-S	51°13′29.4″N 19°09′25.3″E	7.5	2.5	5	19.8 ± 1.6	7.53 ± 0.09	7.88 ± 0.24	0.55^b^ ± 0.09	27.5^b^ ± 15.6	27.75^b^ ± 6.99	8.45 ± 3.07	3.27 ± 1.44	5.18 ± 3.56	1.67^ab^ ± 1.23	0.134 ± 0.04	0.323 ± 0.12
PN	OM-B	51º15′13.9″N 19º22′01.3″E	8.0	1.7	35	18.2 ± 2.0	7.62 ± 0.16	9.28 ± 1.01	0.96^ab^ ± 0.08	8.0^a^ ± 1.41	7.25^ab^ ± 2.06	4.05 ± 2.09	1.52 ± 0.94	2.53 ± 2.38	2.27^ab^ ± 2.16	0.110 ± 0.08	0.240 ± 0.04
WI	OM-B	51°13′24.7″N 19°12′55.0″E	8.2	2.2	20	20.6 ± 1.4	7.61 ± 0.15	7.16 ± 0.67	1.45^a^ ± 0.24	8.25^a^ ± 0.5	7.00^a^ ± 2.45	5.82 ± 2.54	2.16 ± 1.14	3.66 ± 3.04	0.245^a^ ± 0.18	0.144 ± 0.06	0.319 ± 0.24

Abbreviations: OM-B opencast mining Bełchatów, OM-S opencast mining Szczerców, DO dissolved oxygen, SDT – Secchi Disk Transparency, Tot susp – total suspension, In susp – inorganic suspension, Org susp – organic suspension, Chl *a* chlorophyll *a*, TP total phosphorus, TN total nitrogen.

Values with varying superscript are significantly different among reservoirs by Kruskal-Wallis test (*P* ≤ 0.05).

### Chemical composition of suspensions

**Figure 1:**
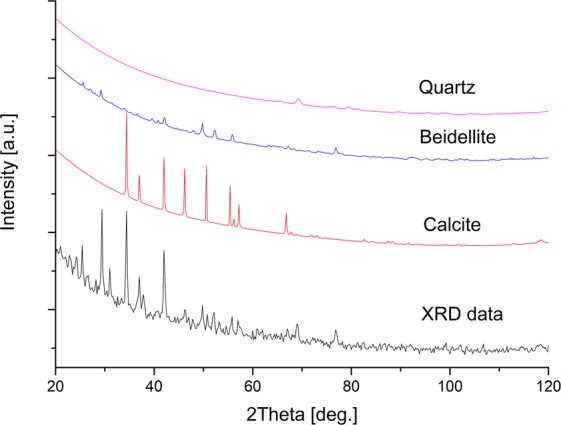
XRD diffractogram of mineral composition of suspensions.

The main minerals found in the suspensions were: beidellite, calcite and quartz (Fig. 1). Precise measurements of the particles show the presence of 14 chemical elements (Table 2), among which carbon and oxygen atoms contributed to the largest weight fractions (29 and 41%), respectively. Silicon was found the third most abundant element (7.3%), the amount of which was correlated with those of Al and Fe (Supplementary Table S1). The highest amount of Si was noted in WI (18.1%), while the lowest in PN (0.14%). The mean fractions of calcium, sodium, and potassium atoms were 6.5, 2.5, and 2.3%, respectively, and correlations of Na and K with Cl appeared significant (Supplementary Table S1). The highest mean fractions of these elements were noted in KA1 (3.73, 3.78, and 4.01%, respectively). The traces of copper atoms were detected in samples: KA1, KU, and PN, whereas titanium in KU only (Table 2).

**Table 2:** Chemical composition (% weight) of suspended particles of the studied reservoirs (mean ± SD).

Reservoir	C	O	Na	Mg	Al	Si	P	S	Cl	K	Ca	Fe	Cu	Ti
CH1	31.72 ± 16.11	40.64 ± 1.46	1.71 ± 0.77	1.77 ± 0.46	1.21 ± 0.96	5.22±2.79	0.06 ± 0.11	1.97 ± 1.40	3.65 ± 4.16	0.90 ± 0.51	7.64 ± 3.93	1.33 ± 0.94	nd	nd
CH2	21.44 ± 13.9	22.51 ± 0.31	3.52 ± 0.60	2.55 ± 1.29	0.97±0.18	3.29 ± 1.07	nd	1.96 ± 1.04	3.46 ± 3.17	4.73 ± 2.68	5.71 ± 2.03	0.88 ± 0.01	nd	nd
KA1	22.49 ± 15.18	33.09 ± 11.45	3.73 ± 3.56	1.53 ± 0.66	2.06 ± 1.88	6.05 ± 1.35	0.38 ± 0.50	1.30 ± 0.27	4.01 ± 3.03	3.78 ± 3.06	3.23 ± 2.20	2.69 ± 3.34	0.48 ± 0.73	nd
KA2	12.44 ± 2.5	42.10 ± 2.03	2.87 ± 0.39	2.57 ± 1.06	2.75 ± 1.34	8.02 ± 3.26	nd	1.99 ± 1.28	2.68 ± 1.71	2.77 ± 1.34	11.01 ± 5.02	1.28 ± 1.01	nd	nd
KA3	33.17 ± 12.7	46.59 ± 3.22	1.64 ± 1.14	0.44 ± 0.17	1.35 ± 0.62	3.25 ± 1.78	0.79 ± 0.23	2.37 ± 0.68	1.74 ± 0.81	0.78 ± 0.03	3.11 ± 1.07	1.30 ± 0.15	nd	nd
KU	36.2 ± 8.2	44.47 ± 4.83	1.55 ± 0.62	1.58 ± 0.97	2.29 ± 1.67	7.25 ± 4.26	nd	0.29 ± 0.02	0.58 ± 0.26	0.86 ± 0.13	2.34 ± 1.26	2.04 ± 0.27	0.15 ± 0.25	0.2 ± 0.02
PN	47.73 ± 6.37	36.83 ± 7.55	1.07 ± 0.94	1.16 ± 0.92	0.91 ± 0.87	2.99 ± 2.59	0.07 ± 0.14	0.98 ± 0.71	0.98 ± 1.06	0.88 ± 0.82	2.56 ± 1.21	0.81 ± 0.48	0.17 ± 0.19	nd
WI	13.33 ± 1.87	34.65 ± 5.65	1.69 ± 1.56	2.84 ± 1.28	4.44 ± 2.77	12.45 ± 6.68	nd	0.91 ± 0.26	1.18 ± 1.19	2.77 ± 1.33	6.87 ± 5.29	2.76 ± 1.57	nd	nd

nd – not detected.

### Geometric and size structure of suspensions

**Figure 2:**
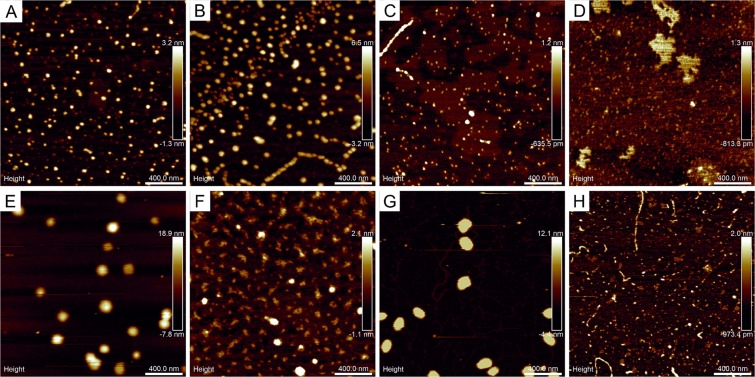
AFM images of nanoparticles in suspension settled on mica substrates (2 µm scan size): (**A**) sample CH1, (**B**) sample CH2, (**C**) sample KA1, (**D**) sample KA2, (**E**) sample KA3, (**F**) sample KU, (**G**) sample PN, (**H**) sample WI.

In general, performed measurements revealed isotropic surface texture of prepared samples, which means that spatial variations of the surface geometry were independent of the direction of observation (Supplementary Fig. S1). The largest nanoparticles seen in AFM approached 100 nm, which were found in samples KA3 and PN (Table 3, Supplementary Fig. S2). Their average surface areas and diameters are significantly larger than in the remaining samples (*P* ≤ 0.05). Fine-grain, regular particles with similar geometric characteristics seen in Fig. 2E,G appeared uniformly dispersed with the areal concentration around 5 µm^−2^. The fraction of nanosized particles (the phase content) reached approximately 5%. In turn, the images of samples CH1, CH2, and KU revealed smaller but dense-packed nanoparticles around 30–50 nm in diameter, surface concentration 60–80 µm^−2^ and the phase content around 10% (Table 3). These nanoparticles were also uniformly distributed, although in CH2 sample also agglomeration processes occurred resulting in formation of linear chains (Fig. 2A,B,F). The smallest nanoparticles were found in samples KA1, KA2, and WI (15–25 nm; Supplementary Fig. S2) with surface concentrations 100–250 µm^−2^. These particles exhibited mutual adhesion that resulted in formation of various geometric patterns: linear chains up to 500 nm long (KA1 and WI) or irregular patches approaching 300–400 nm in diameter (Fig. 2C,D,H). Geometric diversity of aligned nanoparticle forms was connected with large variations in their phase content (4–13%; Table 3). On the other hand, numerical analysis of complexes of microparticles seen in macroscopic images of the samples KA1, KA2, CH2, KU, WI, and PN revealed the presence of single, irregular structures up to 100 µm in size (mean 17 µm), although with small phase content at the micro level (Table 3). In contrast, the macroscopic images of samples CH1 and KA3 exhibited intermediate-size particles (5 µm in diameter) corresponding to large variations in the phase content (0.073 and 1.9%, respectively). Average values of microparticle surface areas and particle diameters in PN, KU, and WI were found higher and significantly different from the others (*P* ≤ 0.05).

**Table 3:** Quantitative and qualitative parameters of suspension particles in individual reservoirs (mean ± SD).

Reservoir	Phase content		Cross section area		Particle size	
	micro (%)	nano (%)	micro (µm^2^)	nano (nm^2^)	micro (µm)^*^	nano (nm)^*^
CH1	0.07	7.8	19 ± 35	848 ± 2122	4.0 ± 3.2	27.2 ± 18.5
CH2	0.34	9.4	51 ± 141	1770 ± 1726	5.1 ± 6.5	43.1 ± 20.0
KA1	1.9	4.5	16 ± 46	533 ± 893	3.9 ± 2.4	23.8 ± 10.5
KA2	6.8	12.9	25 ± 82	384 ± 2863	4.7 ± 3.2	14.1 ± 17.1
KA3	1.9	6.6	18 ± 25	6213 ± 6016	4.1 ± 2.4	73.5 ± 50.7
KU	0.82	11.6	103 ± 265	933 ± 1865	7.4 ± 9.1	27.4 ± 20.9
PN	2.6	4.3	141 ± 368	3574 ± 5567	8.0 ± 11.0	47.6 ± 48.4
WI	0.47	8.8	83 ± 251	334 ± 528	5.9 ± 8.8	17.4 ± 11.1

^*^Statistically significant differences by Friedman’s test (*P* ≤ 0.05).

### Zooplankton composition and diversity

**Figure 3:**
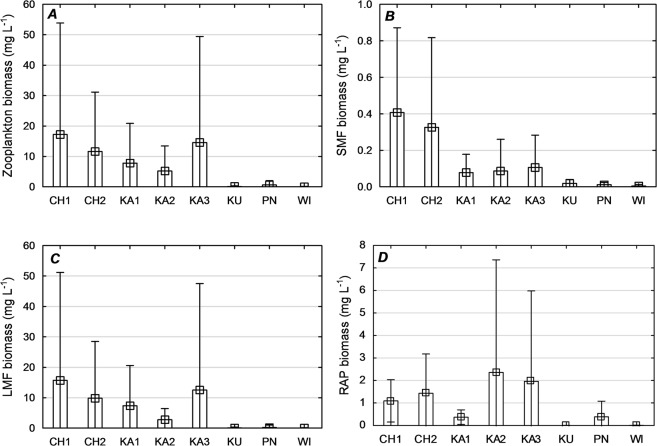
Mean values of zooplankton functional groups biomass (mg L^−1^) in individual reservoirs. Small square: average, rectangle: ± standard error, “swirls”: ± SD.

In the zooplankton of the reservoirs under analysis, 46 taxa were identified, including 35 Rotifera, 6 Copepoda, and 5 Cladocera. The number of species was the highest in CH1 (13–18) and the lowest in KU (3–9). The zooplankton of WI and CH1 was highly diverse (*H*′ = 1.60 and 1.54; *J*′ = 0.734 and 0.551, respectively; Supplementary Table S2). The zooplankton of KA1 was marked by low diversity (*H*′ = 1.29) and evenness (*J*′ = 0.552) index values. The greatest ranges of zooplankton abundance were determined in CH1 (805–2,840 ind. L^−1^). The lowest ranges of zooplankton abundance were determined in WI (9–60 ind. L^−1^). In all of the reservoirs studied, rotifers dominated comprising from 43% (KU) to 95% (PN) of the overall zooplankton abundance. The dominants were *Polyarthra longiremis* (12–63%), *Ascomorpha ovalis* (22–30%), and *Filinia longiseta* (6–8%) and species of the genera *Keratella* (2–15%) and *Synchaeta* (2–12%). Quantitatively, Cladocera contributed from 1.8 (KU) to 25.2% (KA1). Copepoda were represented mainly by larval nauplii forms (4–53%) and copepodites (2–5%; Supplementary Table S2). The greatest faunal similarity of the zooplankton assemblages was noted in CH1 and CH2 (81%), KA1 and PN (63%), and KU and WI (69%; Supplementary Fig. S3). The highest zooplankton biomass was recorded in CH1 (0.71–39.6 mg L^−1^), while the lowest was in WI (0.04–0.16 mg L^−1^). The difference in the mean zooplankton biomass value among the reservoirs was statistically significant (*P* ≤ 0.05; Fig. 3A).

### Zooplankton functional groups

The rotifers *Filinia longiseta*, *Keratella valga*, *K*. *cochlearis*, *K*. *tecta*, and *Hexarthra mira* and copepod nauplii were dominant in the SMF group. The crustaceans *Daphnia cucullata* and *Eubosmina longirostris* and copepodites dominated the LMF group, while the rotifers *Polyarthra longiremis*, *Ascomorpha ovalis*, *Asplanchna priodonta*, and *Synchaeta* spp. dominated the RAP group (Supplementary Table S2). CH1 was characterized by the highest biomass values of small and large microphagous: SMF (0.08–0.62 mg L^−1^), LMF (0.12–37.4 mg L^−1^). Raptorials dominated in KA2 (0.21–5.9 mg L^−1^) and KA3 (0.27–4.78 mg L^−1^). The differences in the mean values of the SMF, LMF, and RAP functional group biomasses among the reservoirs were statistically significant (*P* ≤ 0.05; Fig. 3B–D).

**Figure 4:**
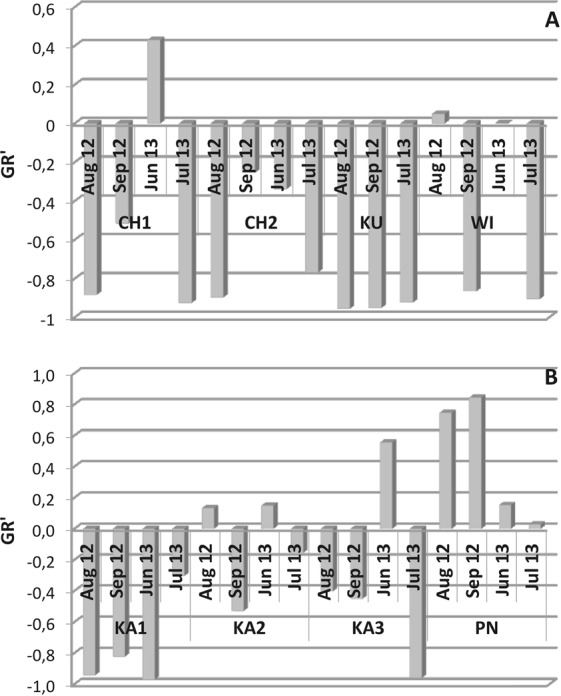
Temporal variations of the trophic ratio (GR′) in individual reservoirs.

**Figure 5:**
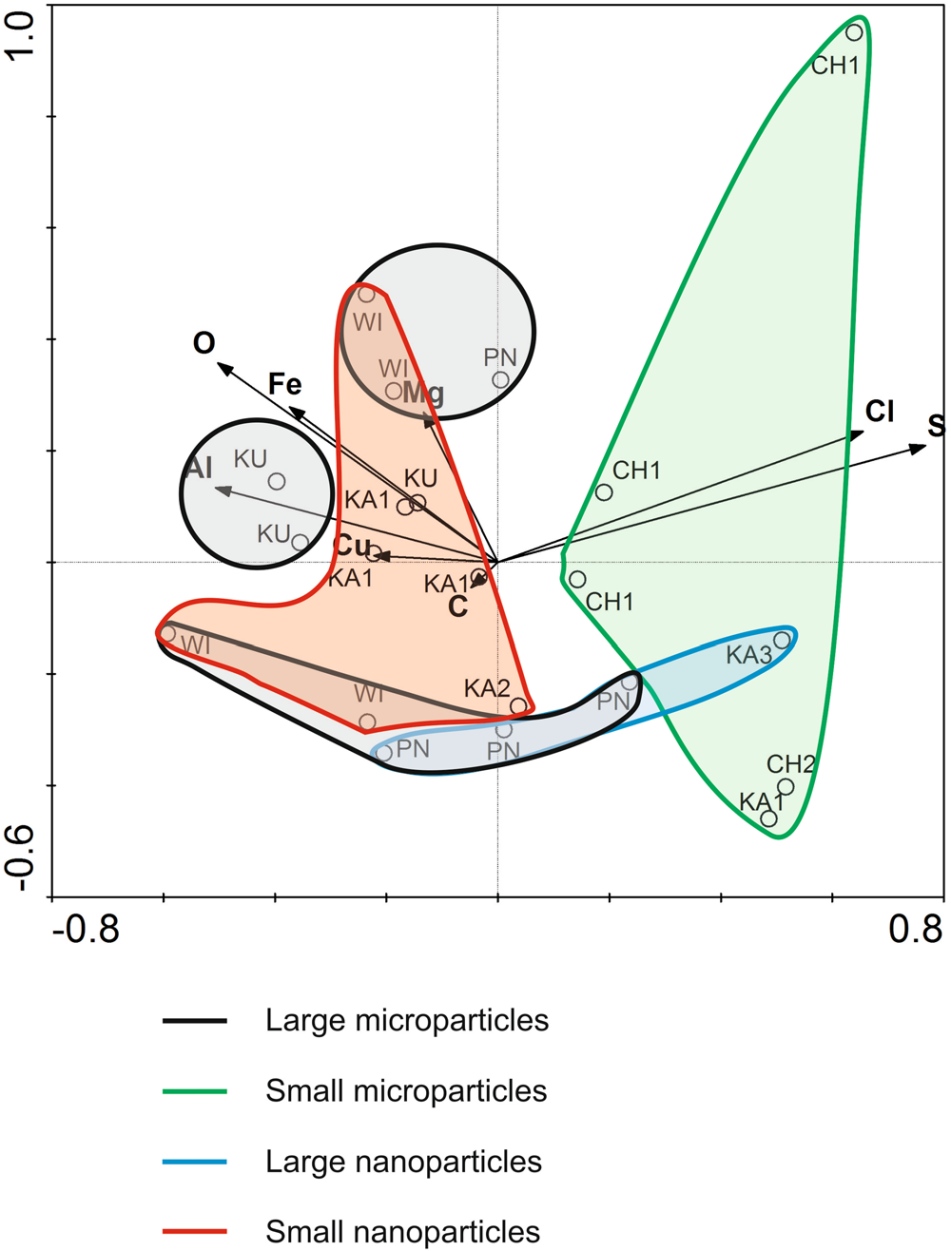
Ordination biplot of redundancy analysis (RDA) for chemical composition of suspension (environmental variables) and samples. Vectors pointing in the same direction indicate a positive correlation, vectors crossing at right angles indicate a near zero correlation, while vectors pointing in opposite direction show a negative correlation.

The mean values and seasonal variations of GR′ were low in KU, KA1, and CH2 (−0.942; −0.769 and −0.603, respectively; Fig. 4A,B). This reflected the permanent domination of microphagous. A high range of seasonal variation in GR′ was observed in KA3 (−0.905–0.555) and CH1 (−0.924–0.430). In turn, the permanent, dominating share of raptorials (mean 72.1%) in PN indicated a positive GR′ value (GR′_mean_ = 0.442; Fig. 5, Supplementary Table S2) for the entire study period.

### Primary gradients affecting zooplankton community

**Figure 6:**
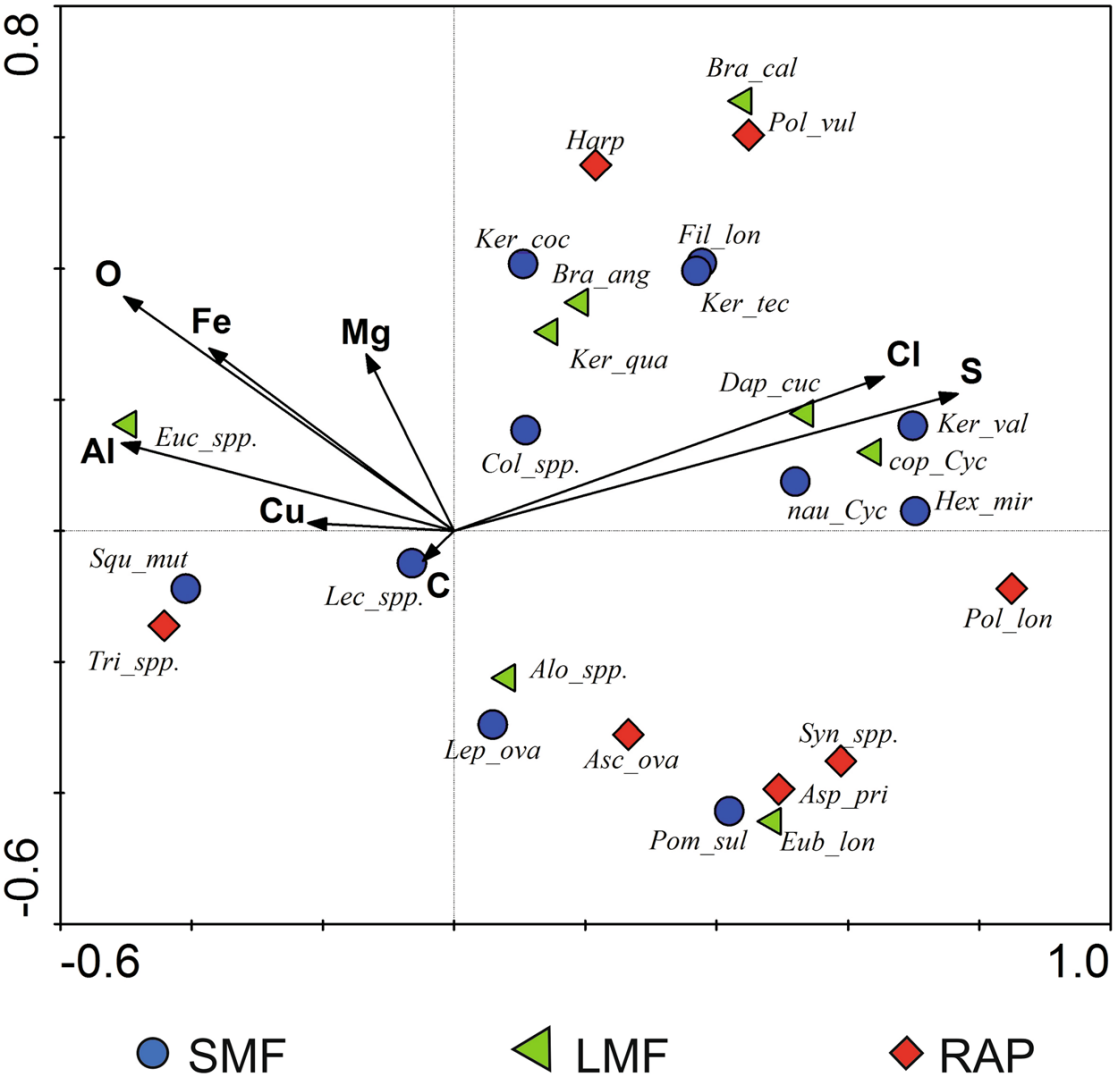
Ordination biplot of redundancy analysis (RDA) for zooplankton communities (species and functional groups) and chemical composition of suspension (environmental variables). Abbreviations used in the figure: *Asc*_*spp*., *Ascomorpha* spp.; *Asp*_*pri*, *Asplanchna priodonta*; *Eub*_*lon*, *Eubosmina longirosrtis*; *Bra*_*ang*, *Brachionus angularis*; *Bra*_*cal*, *Brachionus calyciflorus*; *Cla_juv*, Cladocera juvenile stages; *Col_spp*., *Colurella* spp.; cop_Cyc, copepodite of cyclopoids; *Alo_spp*., *Alona* spp.; *Dap*_*cuc*, *Daphnia cucullata*; *Eub*_*lon*., *Eubosmina longirostris; Euc*_ *spp*., *Euchlanis* spp.; *Fil*_*lon*, *Filinia longiseta*; *Hex_mir*, *Hexarthra mira*; *Harp*, Harpacticoida; *Ker*_*coc*, *Keratella cochlearis; Ker*_*qua*, *Keratella quadrata; Ker*_*tec*, *Keratella tecta*; *Ker_val*, *Keratella valga*; *Lec*_*spp*., *Lecane* spp.; *Lep_ova*, *Lepadella ovalis*; nau_Cyc, nauplii of cyclopoids; *Pol_lon*, *Polyarthra longiremis*; *Pol_vul*, *Polyarthra vulgaris*; *Pom_sul*, *Pompholyx sulcata*; *Syn*_*spp*., *Synchaeta* spp.; *Squ_mut*, *Squatinella mutica*; *Tri_spp*., *Trichocerca* spp.

Variables used in the ordination explained 53% of the total variability of the zooplankton (Supplementary Table S3). The species-environment correlation of all axes became significant in the Monte Carlo permutation test (F = 1.655, *P* < 0.05). The RDAs showed that sulfur (S) related significantly with the zooplankton assemblages. Along the gradient of the first axis, the largest correlation between environmental variables and sample location was for sulfur (S) concentration (*r* = 0.66), along the second axis this was correlated with oxygen (O) concentration (*r* = 0.26). The largest correlation with the third axis was associated with copper (Cu) concentration (*r* = 0.58; Fig. 5). The RDA biplot for species and environmental variables indicated that taxa such as *Keratella valga*, *K*. *tecta*, *Hexarthra mira*, *Daphnia cucullata* and larval forms of Copapoda (nauplii and copepodites) were positively correlated with chlorides and sulphides; are representatives of SMF and LMF groups. Most of RAP species (*Synchaeta* spp., *Asplanchna priodonta*, *Ascomorpha ovalis*, *Polyarthra longiremis*) were negatively correlated with aluminum, iron and magnesium oxides (Fig. 6).

## Discussion

Our results shows that the concentration, size, and chemical composition of the suspension particles can control the diversity and functionality of the zooplankton both directly and indirectly.

The direct reaction of zooplankton upon changes in the concentration of particle suspensions in the water was published previously, see *inter alia*^6,11–13,46–48^. The authors demonstrated that the threshold concentration of suspensions was approximately 50 mg L^−1^ (at a particle size of approximately 1 µm) for Cladocera and above 20 mg L^−1^ for Copepoda, whereas beyond these limits the disruptions in vital parameters were observed. However, no significant impact on Rotifera was observed. Mean concentrations of suspensions in the waters from the reservoirs did not exceed 10 mg L^−1^, with a maximum of 22.3 mg L^−1^ (KA3) and 17.9 mg L^−1^ (KA1).

This could not permanently limited the functioning of any zooplankton group. The reservoirs were dominated by eurytopic Rotifera (*Polyarthra longiremis*, *Ascomorpha ovalis*, *Filinia longiseta*, *Keratella* spp., *Synchaeta* spp.) that tolerate a wide range of environmental parameters and were often reported to be components of artificial, post-mining waters^1,36,49–52^. Note that the concentrations of suspension did not fluctuate enough among the reservoirs to elicit environmental stress, which is important factor for the zooplankton to generate appropriate adaptive mechanisms^37,46^.

Also, the current study did not detect any negative impact of the concentration of suspensions on the productivity level (Chl *a*), and therefore on the deterioration of food conditions for zooplankton^3–5,8^. Abundant populations of Cladocera species, which is the most demanding feeding group, were observed in reservoirs with heavy suspension loads and, at the same time the high productivity. Assuming that inorganic particles provide excellent media for the adsorption of organic substrates, they could be an alternative source of food for Cladocera under low environmental production^4,10,27,53^. Another factor supporting the development of Cladocera in the reservoirs with the highest concentrations of suspensions was water temperature, as reported previously by Goździejewska *et al*.^1^. With increasing concentration of the suspension and color of the water, the temperature increases as well, which stimulates the intensification of transformation in the trophic chain. As a result, the significance of the LMF functional group increased, as demonstrated by Moreira *et al*.^14^. The worst feeding conditions (Chl *a* = 0.245 µg L^−1^) were noted with the lowest parameters of suspended material (2.3–8.2 mg L^−1^; 7 NTU) that were confirmed in WI and that were responsible for the lowest zooplankton abundance and biomass.

The turbidity parameter, expressed in nephelometric turbidity units (NTU), was used to interpret water suspension loads^6,37,47,54,55^. In most of the reservoirs under study, the NTU was found 2–3 times higher than the concentration suspensions (mg L^−1^), while in natural waters, either the inverse proportion between these parameters or a ratio close to 1 is usually observed^46^. Bilotta and Brazier^6^ reported that turbidity depend, *inter alia*, on the size and shape of suspended particles and phytoplankton production. Therefore, high turbidity might not univocally indicate high suspension concentrations.

Previous observations on the direct impact of increased turbidity on zooplankton under natural conditions refer to sudden disruptions in ecosystems. These involved the intense though transient occurrence of particles of various sizes in the water due to atmospheric factors (e.g., sediment resuspension by wind, surface runoff from catchments during heavy precipitation), which usually resulted in stress and elimination of sensitive species^47,54,55^. In the current analysis, turbidity was found to not have a direct impact on any of the zooplankton functional groups. Assuming low primary production, high turbidity values in relation to low measures of suspension concentrations indicate that particles were small^6^.

In addition to microparticles, a significant content of nanometric particles was confirmed in the suspensions. According to previous statements, experiments performed to determine the impact the microparticles have on filtering organisms have usually been done in laboratory conditions and focused on concentrations of suspensions^10–12^. On the other hand, studies on the impact of nanoparticles focus mainly on their high bioactivity^17^, which is determined by the following relationship: smaller particles exhibit larger active surface area resulting in higher bioavailability, which ends up in increased toxicity^28,56^. Experiments demonstrated that the toxicity of nanoparticles of a specific chemical composition (usually artificially synthesized compounds) depends on the biology of the plankton species subjected to the toxicological tests and to the environment of the interactions^22,25,31^.

The chemical composition of the suspensions was found similar to that of natural bedrocks (primarily beidellite clays) usually made from: silicon, aluminum, iron, magnesium, calcium, and potassium oxides and calcium carbonates, in agreement with results published by Ratajczak *et al*.^57^. Nanoparticles appeared to be the dominant part of the mineral fraction of the suspensions responsible for the cycling of the elements^7,9^. With increasing nanoparticle content, the amount of silicon and magnesium in the suspensions also increased. Additionally, the smaller size of the nanoparticles corresponded to higher content of Si, Mg, Al, and Fe atoms in KU, WI, and KA1 samples (Fig. 5). These elements limited most zooplankton species, especially raptorials (Fig. 6). Many studies confirm high reactivity of nanoparticles of aluminum, iron and copper compounds in aquatic ecosystems leading to inhibition of algal growth and increased mortality of zooplankton^30,58,59^ (Cu was detected in suspension particles of reservoirs KA1 and KU). Through accumulation in the trophic chain, they might also exert toxic effects on fish^60^. However, it was found that the harmfulness of iron oxide nanoparticles may effectively reduced by silica and calcium^30^, which in our research are important.

It was shown that the biological effect of nanoparticles depends not only on the chemical structure, but also on the physical properties related to the velocity of particle aggregation. This property determines the response time of an organism whose cell surface was left in contact with the aggregates^61–63^. In our studies the tendency to aggregate the smallest, silicon-rich nanoparticles was observed. This could be the cause of low primary production in the WI reservoir, due to the deposition of silica nanoparticles on the surface of algae cells. Under limited food resources, the participation of all functional groups of zooplankton was similar, which proved that there was a co-existence without competitive elimination.

Another factor that could further limit the zooplankton was the large morphological diversity of nanoparticles (in KA1 and WI) and the large amplitude of size, i.e. the simultaneous occurrence of the of the smallest nano- and the largest microparticles (in KU and WI). Zhang *et al*.^2^ reported that suspended microparticles of natural origin (montmorillonite) are more toxic to *Daphnia magna* than nanostructures of the same composition. Obtained results turned out ambiguous in terms of the influence the largest suspension particles had on Cladocera, however, the low abundance and frequency of Cladocera in KU and WI (1–3 ind. L^−1^) could be certainly traced back to the poor feeding conditions. The above phenomena in KU, KA1, and WI reservoirs resulted in low zooplankton functional diversity (Fig. 4).

The microscale suspension fractions were found responsible for sulfur, chlorine, phosphorus, and sodium cycling. With decreasing diameter of the microparticles the content of these elements in the suspensions increased. These conditions promoted large abundance and diversity of Rotifera and Crustacea and also growth in the biomass of all functional zooplankton groups (Figs 5 and 6), which were confirmed in reservoirs CH1, CH2, and KA3. At the same time, the functionalities of CH1 and KA3 were greater because of multi-directional use of environmental resources by the zooplankton (Fig. 4). Unlike these reservoirs, however, the oldest reservoir under study (PN) was found dominated by raptorials with similar functional traits. The specific feeding conditions most likely determined this diatom species richness^1^, which the RAP group exploited most effectively.

In conclusion, we have shown that differences in zooplankton structure are caused by the factors related to environmental conditions, among which suspension parameters are responsible largely to the functional gradients of the reservoirs under study. The relative amounts of micro- and nanoparticle content and their affinity for specific elements were the parameters that regulated the functional diversity of the zooplankton. It was limited by the most extreme particle sizes in suspensions i.e., the upper range of microphase and the lower range of nanophase. Small microphages were found the least sensitive and/or the quickest to adapt to the spectrum of particle sizes. On the other hand, the population of large microphages was limited by the largest microparticles, whereas that of raptorials mainly by the smallest nanoparticles (Fig. S4). Functionality of the ecosystem was found high when suspension parameters were intermediate, which corresponds to the dominance of the smallest micro- and the largest nanoparticles and a balanced chemical composition. This condition was indicated by the co-existence of all functional zooplankton groups (Fig. S4). In the oldest reservoirs, where the suspensions contained a range of nanoparticles of all sizes and small content of the largest microparticles, the even, multi-directional exploitation of food resources was noted indicating that these ecosystems were highly resistant to disruptions.

The environments of the analyzed reservoirs present a unique opportunity to research *in situ* the impacts suspended micro- and nanoparticles of natural origin have on zooplankton.

However, it should be emphasized that clear demonstration of the effect of suspension particles on zooplankton in environmental studies is very difficult task. Many mechanisms of chemical transformations (e.g., solubility, speciation, and aggregation) and interactions (e.g., adhesion on the surface of living organisms, accumulation inside organisms) and transfer in the food chain should be taken into account. The application of physical research methodology help to identifying the direction and intensity of hydrobiological processes and interpreting them in accordance with the “intermediate disturbance hypothesis”.

## Materials and Methods

### Study area

**Figure 7:**
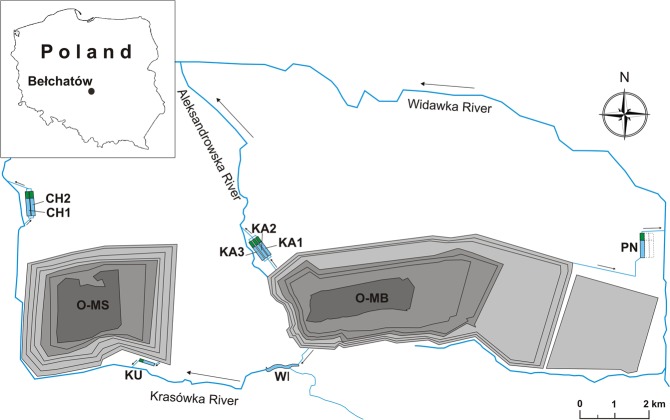
Location of the study area. Abbreviations: O-MB – opencast mining Bełchatów, O-MS – opencast mining Szczerców, chambers of the Chabielice settlement tank complex (CH1, CH2), chambers of the Kamień settlement tank complex (KA1, KA2, KA3), and single-chamber reservoirs: KU, PN, WI.

The study was conducted in eight artificial reservoirs located in the vicinity of the Bełchatów brown coal strip mine. Three of the reservoirs are the chambers of the Kamień settlement tank complex (KA1, KA2, KA3) and two reservoirs are the chambers of the Chabielice settlement tank complex (CH1, CH2), while the Północny (PN), Winek (WI), and Kuźnica (KU) reservoirs are single-chamber reservoirs (Fig. 7). The reservoirs are coupled with the drainage systems of either the Bełchatów or Szczerców open-pit mines (Table 1) and receive waters from different depths of the drainage system that are mixed in variable proportions. Their main function is to reduce suspended matter through sedimentation, but they are also exploited for recreational fishing.

The Winek Reservoir was created by damming the Krasówka River. It is characterized by an elongated shape that is associated with the slight meandering river bed. The immediate vicinity of the shoreline comprises meadows and forests. The basins of the other reservoirs are of a regular, rectangular shape, and the water volumes are similar (approximately 100,000 m^3^). The chamber of each comprises three functional zones: (1) the inflow and the shallow sedimentation zone for the coarsest fraction; (2) the central zone (100 × 400 m) where fine inorganic fractions are deposited and organic compounds are metabolized aerobically; (3) the plant filter zone (approximately 100 m long and 0.25 m deep) where sedimentation concludes. Three chambers of the Kamień settlement tank complex KA and two chambers of CH settlement tank are arranged parallelly to each other, and they are enclosed by embankments, with crowns about 3.5 m wide and slope ratios of 1:2. The embankments are overgrown with meadow vegetation and shrubs.

### Sampling and analytical procedure

Zooplankton were sampled in August and September 2012 (summer/autumn) and in July and June in 2013 (spring/summer). The sampling sites were in the central parts of the basins of each of the eight reservoirs (CH1, CH2, KA1, KA2, KA3, KU, PN, WI). Samples were collected with a 5 L Patalas trap from a depth of approximately 1 m beneath the surface. The sampled material of 20 L was filtered through a plankton net with a mesh size of 30 µm, preserved with Lugol’s solution, and fixed in a 4% formalin solution. The zooplankton was identified under a Zeiss AXIO Imager microscope to the lowest possible taxonomic unit (with the exception of Copepoda juvenile stages) using methods see^64–68^. Quantitative analysis included determining zooplankton abundance using a Sedgewick-Rafter counting chamber. Zooplankton biomass was determined with methods see^69,70^.

The dominance (D)^71^, diversity (Shannon’s index, *H*′), and species evenness (Pielou’s index, *J*′) were analysed. MVSP 3.22 software was used to analyze the taxonomic differentiation and similarity of the zooplankton communities (Bray-Curtis index)^72^.

The zooplankton species were classified to the three groups based on their functional traits of feeding strategy and body size: small microphagous (SMF), large microphagous (LMF), and raptorials (RAP). The methods see^14,44,73,74^ were used to classify the species to trophic groups. The trophic groups ratio (GR′)^73,75^ was used to characterize the zooplankton trophic dynamics in the reservoirs studied.

The GR′ was calculated with the formula:

$$G{\rm{R}}^{\prime} =\sum ({\rm{Raptorial}}\,{\rm{biomass}}-{\rm{Microphagous}}\,{\rm{biomass}})/\sum ({\rm{Total}}\,{\rm{Zooplankton}}\,{\rm{biomass}})$$

The values of GR′ range from −1 to 1. Values < 0 indicate the dominance of microphagous, and values > 0 indicate a dominance of raptorial feeders.

The following physico-chemical parameters were analysed at zooplankton sampling sites at each sampling event: temperature (T, °C), water pH, Secchi Disk Transparency (SDT, m), and dissolved oxygen (DO, mg L^−1^). All physico-chemical parameters were measured using a YSI 6600 V2 Multi-Parameter Water Quality Sonde. Water samples were also collected during each sampling event for laboratory analyses of water color (Hazen), turbidity (NTU), total nitrogen (TN), total phosphorous (TP), and chlorophyll *a* (Chl *a*). The total concentration of suspended matter (Tot susp, mg L^−1^) as well as the organic (Org susp, mg L^−1^) and inorganic (In susp, mg L^−1^) fractions were determined. The hydrochemical analyses were conducted in accordance with APHA guidelines^76^ (Table 1).

The surface structure of particles in the suspensions at the micro- and nanolevels were studied by means of the AFM (Atomic Force Microscopy) method using Multimode 8 instrument with Nanoscope V controller (Bruker), equipped additionally with a small digital camera (approx. 500 × magnification) used to record macroscopic images. The suspensions were investigated in the form of dry sediments. To this end, single droplets of the suspensions under study were first transferred onto mica substrates and left for 5 min to allow the particles to adhere to the substrates. Then, excessive water amount was blown with atmospheric air and the samples were left for another 15 min to get rid of the remaining moisture. In order to obtain nanoscale images, AFM measurements were carried out in a tapping mode under atmospheric conditions. The scans were made using NSG11-B scanning probe (NT-MDT) with the radius 5 nm, force constant 5 N/m, and Au reflective backside coating to increase reflection of the laser beam. The length of the square scan area was 2 µm with 256 steps along each scan axis. The spatial characteristics of the surface texture of the samples were determined according to the procedure described elsewhere^18–21^. The chemical composition of suspended particles was determined with scanning electron microscopy (SEM) and energy dispersive X-ray spectroscopy (EDS). The measurements were conducted with a scanning electron microscope JSM-6610LV (Jeol) and Oxford EDS microanalyzer.

### Statistical procedures

Non-parametric analysis of variance was applied to assess the general differences in suspension parameters in the water and in the parameters determined for the zooplankton among the reservoirs (Statistica 13.0 for Windows, Statsoft, Tulsa). The results were processed by ANOVA using the non-parametric Kruskal-Wallis and Friedman’s tests to determine statistically significant differences among reservoirs in water and suspension parameters, and zooplankton functional structure (*P* ≤ 0.05). Correlation coefficients were calculated with Spearman’s rank correlation coefficient (*P* ≤ 0.05). Detrended correspondence analysis (DCA) was performed on samples in CANOCO 4.56^77^. To reduce the dominating influence of abundant taxa in the multivariate analysis, and abundance data of zooplankton were log (n + 1) transformed^78^.

Environmental variables were analysed for redundancy using Pearson’s correlation. If two variables were highly correlated (r > 0.6 or r < −0.6), the variable which showed the higher overall mean correlation was excluded from further analyses. Detrended Correspondence Analysis (DCA) was used to determine if RDA (Redundancy Analysis) or Canonical Correspondence Analysis (CCA) would be appropriate to evaluate associations between chemical composition of suspended particles and zooplankton abundance.

The DCA ordination gradient was shorter than three standard deviations (1.59 SD), which implied that the linear method was appropriate for the data^79^. The significance of each environmental variable was tested using redundancy analysis (RDA) in an ordination constrained to each suspension-chemistry variable, performed with CANOCO using 499 unrestricted Monte Carlo permutations (reduced model). Backwards selection was conducted and included only environmental variables that were non-collinear (variance inflation factors <10). The automatic forward selection procedure^77^ was used to select the contribution of environmental variables in the explanation of the species data set.

Redundancy analysis was performed for 24 zooplankton taxa and two larval stages of Copepoda (share >2%), and eight environmental variables of suspension. Among all 13 variables (Table 2), Na, Si, P, K, and Ca were not included in the analysed dataset since they were strongly correlated with the variables selected. The variance inflation factor (VIF) of environmental variables included in the analysis displayed very low values and did not exceed the threshold of >8.

## Supplementary information

Supplementary information
