# Supplementary material for: Effects of suspended micro- and nanoscale particles on zooplankton functional diversity of drainage system reservoirs at an open-pit mine
Source: Sci Rep. 2019 Nov 6;9:16113. doi: 10.1038/s41598-019-52542-6 (PMC6834659; doi:10.1038/s41598-019-52542-6)
Supplement: Supplementary file 1 — Supplementary information [file 41598_2019_52542_MOESM1_ESM.docx]

Supplementary Material

**TITLE:**

Effects of suspended micro- and nanoscale particles on zooplankton functional diversity of drainage system reservoirs at an open-pit mine

**Authors:**

**Anna Maria Goździejewska, Monika Gwoździk, Sławomir Kulesza, Mirosław Bramowicz & Jacek Koszałka**

**Table S1** Spearman correlation coefficients between water and suspension parameters and zooplankton structure.

|  | **Chl a** | **SDT** | **Turb** | **Color** | **In Sus** | **Org Sus** | **Tot Sus** | **PCM** | **PCN** | **PSM** | **PSN** | **C** | **O** | **Na** | **Mg** | **Al** | **Si** | **P** | **S** | **Cl** | **K** | **Ca** | **Fe** | **Cu** | **SMF** | **LMF** | **RAP** | **Bio** | **Abu** |
| --- | --- | --- | --- | --- | --- | --- | --- | --- | --- | --- | --- | --- | --- | --- | --- | --- | --- | --- | --- | --- | --- | --- | --- | --- | --- | --- | --- | --- | --- |
| Chl a (µg L^-1^) |  |  |  |  |  |  |  |  |  |  |  |  |  |  |  |  |  |  |  |  |  |  |  |  |  |  |  |  |  |
| SDT (m) | - |  |  |  |  |  |  |  |  |  |  |  |  |  |  |  |  |  |  |  |  |  |  |  |  |  |  |  |  |
| Turb (NTU) | **0.53** | **-0.75** |  |  |  |  |  |  |  |  |  |  |  |  |  |  |  |  |  |  |  |  |  |  |  |  |  |  |  |
| Color (HAZEN) | - | **-0.93** | **0.87** |  |  |  |  |  |  |  |  |  |  |  |  |  |  |  |  |  |  |  |  |  |  |  |  |  |  |
| In Sus (mg L^-1^) | - | **-0.58** | **0.82** | **0.78** |  |  |  |  |  |  |  |  |  |  |  |  |  |  |  |  |  |  |  |  |  |  |  |  |  |
| Org Sus (mg L^-1^) | - | **-0.65** | - | **0.64** | - |  |  |  |  |  |  |  |  |  |  |  |  |  |  |  |  |  |  |  |  |  |  |  |  |
| Tot Sus (mg L^-1^) | **0.62** | **-0.77** | **0.89** | **0.83** | **0.62** | **0.76** |  |  |  |  |  |  |  |  |  |  |  |  |  |  |  |  |  |  |  |  |  |  |  |
| PCM (%) | - | - | - | - | - | - | - |  |  |  |  |  |  |  |  |  |  |  |  |  |  |  |  |  |  |  |  |  |  |
| PCN (%) | - | - | **0.55** | **0.52** | **0.74** | **0.57** | **0.64** | - |  |  |  |  |  |  |  |  |  |  |  |  |  |  |  |  |  |  |  |  |  |
| PSM (µm) | **-0.69** | - | - | - | - | **-0.59** | - | - | - |  |  |  |  |  |  |  |  |  |  |  |  |  |  |  |  |  |  |  |  |
| PSN (nm) | - | - | - | - | **-0.52** | - | - | - | - | - |  |  |  |  |  |  |  |  |  |  |  |  |  |  |  |  |  |  |  |
| C (% W) | - | - | - | - | - | - | - | - | **-0.55** | - | **0.74** |  |  |  |  |  |  |  |  |  |  |  |  |  |  |  |  |  |  |
| O (% W) | **-0.59** | - | - | - | - | - | - | - | - | - | - | - |  |  |  |  |  |  |  |  |  |  |  |  |  |  |  |  |  |
| Na (% W) | **0.81** | - | - | - | - | - | - | - | - | **-0.67** | - | **-0.71** | **-0.53** |  |  |  |  |  |  |  |  |  |  |  |  |  |  |  |  |
| Mg (% W) | - | - | - | - | - | - | - | - | **0.69** | - | **-0.79** | - | - | - |  |  |  |  |  |  |  |  |  |  |  |  |  |  |  |
| Al (% W) | - | - | - | - | - | - | - | - | - | - | **-0.74** | **-0.81** | **0.62** | - | - |  |  |  |  |  |  |  |  |  |  |  |  |  |  |
| Si (% W) | - | - | - | - | **0.62** | - | - | - | **0.64** | - | **-0.90** | **-0.67** | - | - | **0.76** | **0.88** |  |  |  |  |  |  |  |  |  |  |  |  |  |
| P (% W) | - | - | - | - | **-0.51** | - | - | - | **-0.84** | **-0.53** | **0.51** | - | - | - | -**0.85** | - | **-0.63** |  |  |  |  |  |  |  |  |  |  |  |  |
| S (% W) | **0.57** | - | - | - | - | - | - | - | - | **-0.67** | - | - | - | - | - | - | - | - |  |  |  |  |  |  |  |  |  |  |  |
| Cl (% W) | **0.83** | - | - | - | - | - | - | - | - | **-0.81** | - | - | **-0.53** | **0.88** | - | - | - | - | **0.53** |  |  |  |  |  |  |  |  |  |  |
| K (% W) | **0.62** | - | - | - | - | - | - | - | - | - | **-0.50** | **-0.71** | **-0.71** | **0.86** | **0.57** | - | - | - | - | **0.64** |  |  |  |  |  |  |  |  |  |
| Ca (% W) | - | - | - | - | - | - | - | - | - | - | **-0.53** | **-0.79** | - | - | **0.67** | - | - | - | **0.57** | - | - |  |  |  |  |  |  |  |  |
| Fe (% W) | - | - | - | - | - | - | - | - | - | - | **-0.62** | - | - | - | - | **0.69** | **0.69** | - | - | - | - | - |  |  |  |  |  |  |  |
| Cu (% W) | - | - | - | - | - | - | - | - | **-0.52** | - | - | **0.52** | - | - | **-0.52** | - | - | - | **-0.52** | - | - | **-0.76** |  |  |  |  |  |  |  |
| SMF (µg L^-1^) | - | - | - | - | - | - | - | **-0.55** | - | **-0.57** | - | - | **-0.50** | - | - | **-0.53** | - | - | **0.74** | **0.64** | - | - | - |  |  |  |  |  |  |
| LMF (µg L^-1^) | **0.64** | - | - | - | - | - | - | **-0.52** | - | **-0.69** | - | - | - | **0.79** | - | -0.60 | - | - | **0.79** | **0.69** | **0.55** | - | - | - | **-** |  |  |  |  |
| RAP (µg L^-1^) | **0.50** | - | - | - | - | - | - | - | - | **~~-~~** | **-** | - | - | - | - | **-0.76** | **-0.67** | - | **0.93** | **0.62** | - | **0.59** | **-0.62** | **0.55** | **0.60** | **-** |  |  |  |
| Bio (µg L^-1^) | **0.57** | - | - | - | - | - | - | - | - | **-0.69** | - | - | - | **0.53** | - | **-0.60** | **-0.52** | - | **0.79** | **0.69** | - | - | - | - | **-** | **0.66** | **0.93** |  |  |
| Abu (ind. L^-1^) | **0.62** | - | - | - | - | - | - | - | - | **0.50** | - | - | - | - | - | - | - | - | - | - | - | - | **-0.50** | - | **0.85** | **0.83** | **0.88** | **0.98** |  |
| *H’* | **-0.56** | - | - | - | - | - | - | **-0.69** | **0.53** | - | - | - | - | - | **0.71** | - | **0.51** | **-0.71** | - | - | - | - | - | **-0.58** | - | - | - | - | - |

Abbreviations: Chl *a* - Chlorophyll *a*, SDT – Secchi Disk Transparency, Turb – Turbidity, In sus – Inorganic suspension, Org sus – Organic suspension, Tot sus – Total suspension, PCM – Phase content micro, PCN – Phase content nano, PSM – Particle size micro, PSN – Particle size nano, SMF – Small microphagous, LMF – Large microphagous, RAP – Raptorials, Bio – Total zooplankton biomass, Abu – Total zooplankton abundance, *H’* – Shannon’s biodiversity index. Bold values indicate significance *P* < 0.05; - – no siginificant.

**Table S2** Qualitative and quantitative structure of zooplankton based on dominating and frequent taxa in the individual reservoirs (mean ± SD).

| **TAXA** | Group | | **CH1** | |  | **CH2** | |  | **KA1** | |  | **KA2** | |  | **KA3** | |  | **KU** | |  | **PN** | |  | **WI** | |
| --- | --- | --- | --- | --- | --- | --- | --- | --- | --- | --- | --- | --- | --- | --- | --- | --- | --- | --- | --- | --- | --- | --- | --- | --- | --- |
|  | Tro | Eco | **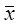** | ±SD |  | **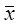** | ±SD |  | **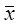** | ±SD |  | **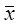** | ±SD |  | **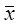** | ±SD |  | **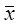** | ±SD |  | **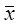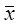** | ±SD |  | **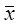** | ±SD |
| **Rotifera (ind. L**^-1^**)** |  |  | **1346** | **910** |  | **1787** | **1167** |  | **305** | **330** |  | **927** | **691** |  | **1624** | **1749** |  | **15** | **7** |  | **277** | **218** |  | **18** | **20** |
| *Ascomorpha ovalis* (Bergendal, 1892) | RAP | *eu* |  |  |  | 24 | 43 |  | 113** | 199 |  | 83* | 123 |  | 450** | 900 |  |  |  |  | 87** | 81 |  |  |  |
| *Asplanchna priodonta* Gosse, 1850 | RAP | *eu* | 14 | 24 |  | 20 | 33 |  | 3 | 3 |  | 89* | 101 |  | 54 | 98 |  |  |  |  | 9 | 10 |  |  |  |
| *Brachionus angularis* Gosse, 1851 | LMF | *eu* | 7 | 7 |  | 1 | 2 |  |  |  |  | 1 | 1 |  |  |  |  | 2* | 3 |  |  |  |  | <1 | 1 |
| *Brachionus calyciflorus* Pallas,1766 | LMF | *eu* | 17 | 34 |  |  |  |  |  |  |  |  |  |  |  |  |  |  |  |  |  |  |  |  |  |
| *Brachionus diversicornis* (Daday, 1883) | LMF | *eu* |  |  |  |  |  |  |  |  |  |  |  |  |  |  |  |  |  |  | 1 | 2 |  |  |  |
| *Cephalodella* spp. | RAP | *ps* |  |  |  |  |  |  |  |  |  |  |  |  |  |  |  |  |  |  |  |  |  | <1 | 1 |
| *Colurella* spp. | SMF | *ps* | 1 | 2 |  |  |  |  |  |  |  |  |  |  |  |  |  |  |  |  | 1 | 3 |  | <1 | 1 |
| *Euchlanis* spp. | LMF | *ps* |  |  |  |  |  |  |  |  |  |  |  |  |  |  |  | 0 | 1 |  |  |  |  | 1 | 1 |
| *Filinia longiseta* (Ehrenberg, 1834) | SMF | *eu* | 107* | 111 |  | 3 | 3 |  |  |  |  | 36 | 72 |  | 2 | 3 |  | 3* | 4 |  | 1 | 1 |  | 2* | 2 |
| *Hexarthra mira* (Hudson, 1871) | SMF | *eu* | 14 | 12 |  | 41 | 53 |  | 4 | 8 |  | 17 | 31 |  |  |  |  |  |  |  |  |  |  |  |  |
| *Keratella cochlearis* (Gosse, 1851) | SMF | *eu* | 27 | 17 |  | 45 | 81 |  | 1 | 3 |  | 3 | 4 |  | 1 | 2 |  | 2* | 2 |  | 1 | 1 |  | 1 | 1 |
| *Keratella quadrata* (Müller, 1786) | LMF | *eu* | 11 | 10 |  | 3 | 3 |  |  |  |  |  |  |  |  |  |  | 1 | 1 |  |  |  |  | 1 | 1 |
| *Keratella tecta* (Gosse, 1851) | SMF | *eu* | 72 | 75 |  | 29 | 37 |  |  |  |  | 1 | 1 |  |  |  |  | 1 | 1 |  | 29** | 45 |  | 1 | 1 |
| *Keratella testudo* (Ehrenberg, 1832) | SMF | *eu* |  |  |  |  |  |  |  |  |  |  |  |  |  |  |  |  |  |  | 1 | 2 |  |  |  |
| *Keratella valga* (Ehrenberg, 1834) | SMF | *eu* | 183** | 111 |  | 93 | 105 |  | 2 | 3 |  | 29 | 51 |  | 44 | 40 |  | 2* | 3 |  |  |  |  | 3** | 7 |
| *Lecan*e spp. | SMF | *ps/eu* | 1 | 1 |  | 1 | 1 |  | 1 | 1 |  | 1 | 2 |  |  |  |  | <1 | 1 |  | <1 | 1 |  | 1 | 1 |
| *Lepadella ovalis* (Müller, 1786) | SMF | *ps* |  |  |  | 1 | 2 |  |  |  |  | 1 | 1 |  |  |  |  |  |  |  | 1 | 1 |  | 1 | 1 |
| *Mytilina mucronata* (Müller, 1773) | LMF | *ps* |  |  |  |  |  |  |  |  |  | 1 | 1 |  |  |  |  |  |  |  |  |  |  |  |  |
| *Notholca* spp. | SMF | *eu* |  |  |  | 1 | 1 |  |  |  |  |  |  |  |  |  |  |  |  |  | <1 | 1 |  |  |  |
| *Polyarthra longiremis* Carlin, 1943 | RAP | *eu/li* | 799** | 799 |  | 1424** | 1158 |  | 168** | 278 |  | 635** | 515 |  | 1042** | 898 |  | 4** | 4 |  | 99** | 142 |  | 6** | 8 |
| *Polyarthra vulgaris* Carlin, 1943 | RAP | *eu/li* | 45 | 71 |  | 66 | 132 |  |  |  |  |  |  |  |  |  |  |  |  |  |  |  |  |  |  |
| *Pompholyx sulcata* Hudson, 1885 | SMF | *eu* | 1 | 1 |  | 32 | 62 |  | 5 | 10 |  | 2 | 4 |  | 4 | 5 |  |  |  |  | 12 | 9 |  | 1 | 2 |
| *Squatinella mutica* (Ehrenberg, 1832) | SMF | *ps* |  |  |  |  |  |  |  |  |  |  |  |  |  |  |  |  |  |  |  |  |  | <1 | 1 |
| *Synchaeta* spp. | RAP | *eu/li* | 46 | 33 |  | 4 | 6 |  | 9 | 11 |  | 31 | 56 |  | 28 | 12 |  |  |  |  | 34** | 64 |  |  |  |
| *Testudinella patina* (Herman, 1783) | SMF | *eu/li* |  |  |  |  |  |  |  |  |  |  |  |  |  |  |  |  |  |  | 1 | 1 |  |  |  |
| *Trichocerca* spp. | RAP | *eu/li* |  |  |  |  |  |  |  |  |  | 1 | 1 |  |  |  |  | 1 | 1 |  | 1 | 1 |  | 1 | 1 |
| *Trichotria pocillum* (Müller, 1776) | SMF | *eu* |  |  |  |  |  |  |  |  |  | <1 | 1 |  |  |  |  |  |  |  |  |  |  |  |  |
| **Cladocera (ind. L^-1^)** |  |  | **179** | **221** |  | **134** | **80** |  | **87** | **73** |  | **88** | **80** |  | **127** | **151** |  | **1** | **1** |  | **5** | **7** |  | **2** | **3** |
| *Alona* spp. | LMF | *li* |  |  |  |  |  |  |  |  |  | 1 | 1 |  | 1 | 1 |  |  |  |  |  |  |  | 0 | 1 |
| *Bosmina longirostris* (Schoedler, 1866) | LMF | *eu* | 33 | 61 |  | 46 | 40 |  | 14 | 14 |  | 66* | 81 |  | 17 | 15 |  |  |  |  | 3 | 4 |  | 1 | 3 |
| *Chydorus sphaericus* (Müller, 1785) | LMF | *eu* |  |  |  |  |  |  |  |  |  |  |  |  | 1 | 1 |  |  |  |  |  |  |  |  |  |
| *Daphnia cucullata* Sars, 1862 | LMF | *eu* | 146* | 169 |  | 88 | 89 |  | 71** | 65 |  | 21 | 20 |  | 108* | 143 |  | 1 | 1 |  | 2 | 4 |  | 0 | 1 |
| *Diaphanosoma brachyurum* (Liévin, 1848) | LMF | *eu* |  |  |  |  |  |  | 1 | 1 |  |  |  |  | 1 | 1 |  |  |  |  |  |  |  |  |  |
| *Leptodora kindtii* (Focke, 1844) | RAP | *eu* |  |  |  |  |  |  | 1 | 1 |  |  |  |  | 0 |  |  |  |  |  |  |  |  |  |  |
| **Copepoda (ind. L^-1^)** |  |  | **384** | **259** |  | **349** | **336** |  | **112** | **62** |  | **67** | **45** |  | **130** | **93** |  | **21** | **9** |  | **5** | **3** |  | **4** | **3** |
| *Cryptocyclops bicolor* (Sars, 1863) | RAP | *li* |  |  |  |  |  |  | 1 | 1 |  | 1 | 2 |  | 1 | 2 |  |  |  |  |  |  |  |  |  |
| *Cyclops strenuus* Fischer, 1851 | RAP | *eu* |  |  |  |  |  |  | 2 | 2 |  | 1 | 1 |  |  |  |  |  |  |  |  |  |  |  |  |
| *Eucyclops speratus* (Lilljeborg, 1901) | RAP | *li* |  |  |  |  |  |  |  |  |  |  |  |  |  |  |  |  |  |  | <1 | 1 |  |  |  |
| *Thermocyclops crassus* (Fischer, 1853) | RAP | *eu* | 5 | 5 |  | 6 | 10 |  | 1 | 1 |  | 2 | 2 |  | 1 | 1 |  |  |  |  |  |  |  |  |  |
| Harpacticoida | RAP | *ps* | 1 | 2 |  |  |  |  |  |  |  |  |  |  |  |  |  |  |  |  |  |  |  | <1 | 1 |
| nauplii | SMF | *eu* | 290** | 209 |  | 240** | 203 |  | 87** | 46 |  | 41 | 21 |  | 104* | 75 |  | 19** | 11 |  | 4 | 2 |  | 3** | 2 |
| copepodites | LMF | *eu* | 89* | 77 |  | 103* | 130 |  | 22 | 19 |  | 22 | 27 |  | 25 | 17 |  | 2* | 2 |  | 1 | 1 |  | 1 | 1 |
| **Abundance of zooplankton (ind. L^-1^)** |  |  | **1909** | **1390** |  | **2270** | **1583** |  | **504** | **465** |  | **1082** | **816** |  | **1881** | **1993** |  | **37** | **17** |  | **287** | **228** |  | **24** | **26** |
| Rotifera (%) |  |  | 72.3 | 24.4 |  | 79.9 | 15.6 |  | 44.8 | 43.1 |  | 84.2 | 9.7 |  | 69.1 | 27.9 |  | 42.9 | 19.5 |  | 94.8 | 4.8 |  | 74.6 | 15.7 |
| Cladocera (%) |  |  | 8.6 | 12.1 |  | 6.3 | 3.5 |  | 25.2 | 29.0 |  | 7.5 | 5.9 |  | 9.0 | 8.6 |  | 1.8 | 3.0 |  | 2.5 | 3.4 |  | 5.3 | 6.1 |
| Copepoda (%) |  |  | 19.1 | 14.3 |  | 13.8 | 13.8 |  | 29.8 | 25.3 |  | 8.3 | 8.5 |  | 21.9 | 27.1 |  | 55.3 | 21.3 |  | 2.7 | 1.7 |  | 20.1 | 12.9 |
| **Biomass of zooplankton (mg L^-1^)** |  |  | **17.21** | **18.33** |  | **11.61** | **9.78** |  | **7.81** | **6.53** |  | **5.21** | **4.12** |  | **14.57** | **17.44** |  | **0.106** | **0.116** |  | **0.614** | **0.722** |  | **0.047** | **0.076** |
| SMF (%) |  |  | 7.0 | 6.3 |  | 3.5 | 2.0 |  | 1.6 | 1.2 |  | 1.9 | 0.9 |  | 1.6 | 1.3 |  | 41.1 | 45.3 |  | 5.2 | 4.9 |  | 29.0 | 29.3 |
| LMF (%) |  |  | 66.7 | 36.1 |  | 74.7 | 17.3 |  | 86.4 | 16.4 |  | 53.1 | 16.5 |  | 64.1 | 31.8 |  | 56.1 | 45.2 |  | 22.8 | 21.2 |  | 42.5 | 30.5 |
| RAP (%) |  |  | 26.3 | 31.5 |  | 21.8 | 15.8 |  | 12.0 | 15.5 |  | 44.9 | 15.9 |  | 34.3 | 31.6 |  | 2.9 | 0.9 |  | 72.1 | 20.6 |  | 28.5 | 26.3 |
| Number of species (ind.) |  |  | 16 | 2 |  | 15 | 2 |  | 11 | 2 |  | 14 | 2 |  | 12 | 1 |  | 9 | 2 |  | 11 | 2 |  | 10 | 6 |
| Pielou’s eveness index, J’ |  |  | 0.551 | 0.041 |  | 0.508 | 0.088 |  | 0.552 | 0.053 |  | 0.516 | 0.035 |  | 0.543 | 0.08 |  | 0.655 | 0.143 |  | 0.558 | 0.052 |  | 0.734 | 0.095 |
| Shannon’s biodiversity index, *H'* |  |  | 1.54 | 0.15 |  | 1.36 | 0.18 |  | 1.29 | 0.06 |  | 1.35 | 0.06 |  | 1.33 | 0.15 |  | 1.40 | 0.28 |  | 1.33 | 0.08 |  | 1.60 | 0.18 |
| Trophic groups ratio, GR' |  |  | -0.473 | 0.629 |  | -0.603 | 0.192 |  | -0.769 | 0.191 |  | -0.491 | 0.197 |  | -0.476 | 0.340 |  | -0.942 | 0.370 |  | 0.442 | 0.339 |  | -0.429 | 0.357 |

Denotations: *eu* – eurytopic, *ps* – psammonic, *li* – littoral taxa; SMF - small microphagous, LMF – large microphagous, RAP – raptorials. ** - eudominant (≥ 10%), * - dominant (5–9.9%).

**Table S3** Summary statistics for the first four axes of RDA of zooplankton data from analyzed reservoirs in June/July (spring/summer season) and August/September (summer/autumn season) in 2012-2013

| **AXES** | **1** | **2** | **3** | **4** | **Total**  **variance** |
| --- | --- | --- | --- | --- | --- |
| Eigenvalues: | 0.264 | 0.119 | 0.109 | 0.058 | 1.000 |
| Species-envir. correl: | 0.945 | 0.948 | 0.947 | 0.887 |  |
| Cumulative % variance |  |  |  |  |  |
| of species data: | 26.4 | 38.2 | 49.2 | 55.0 |  |
| of species-envir. relation: | 36.1 | 52.4 | 67.4 | 75.4 |  |
|  |  |  |  |  |  |
| Sum of all eigenvalues |  |  |  |  | 1.000 |
| Sum of all canonical eigenvalues |  |  |  |  | 0.729 |

**
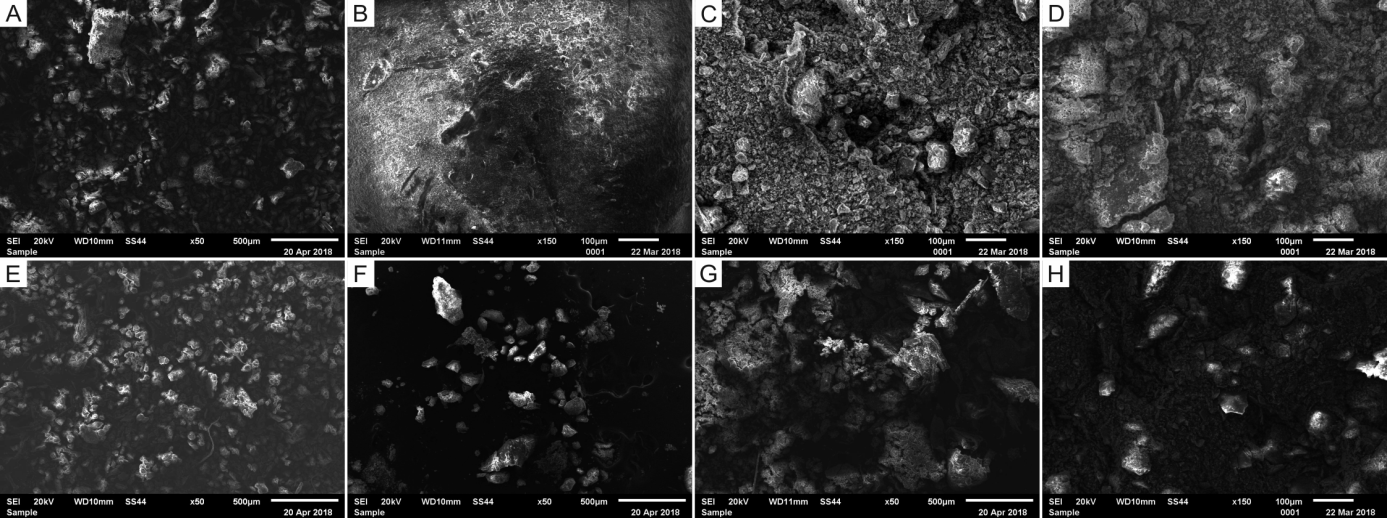
**

**Figure S1** SEM images of sediments from the suspension in the individual reservoirs: (A) sample CH1, (B) sample CH2, (C) sample KA1, (D) sample KA2, (E) sample KA3, (F) sample KU, (G) sample PN, (H) sample WI.

**
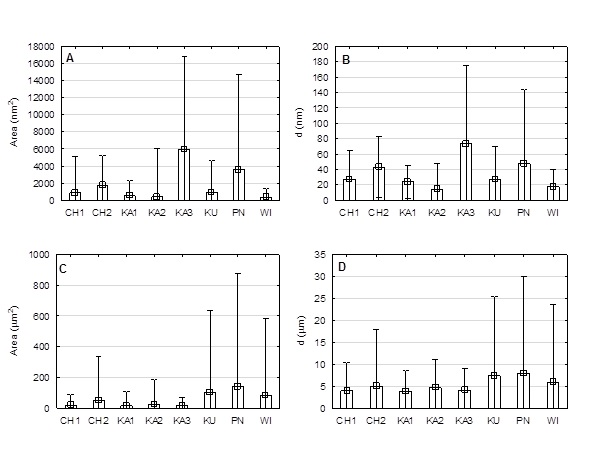
**

**Figure. S2** Mean values of the area and diameter of nano (A, B) and micro (C, D) particles in individual reservoirs. Small square: average, “swirls”: ±SD.

**
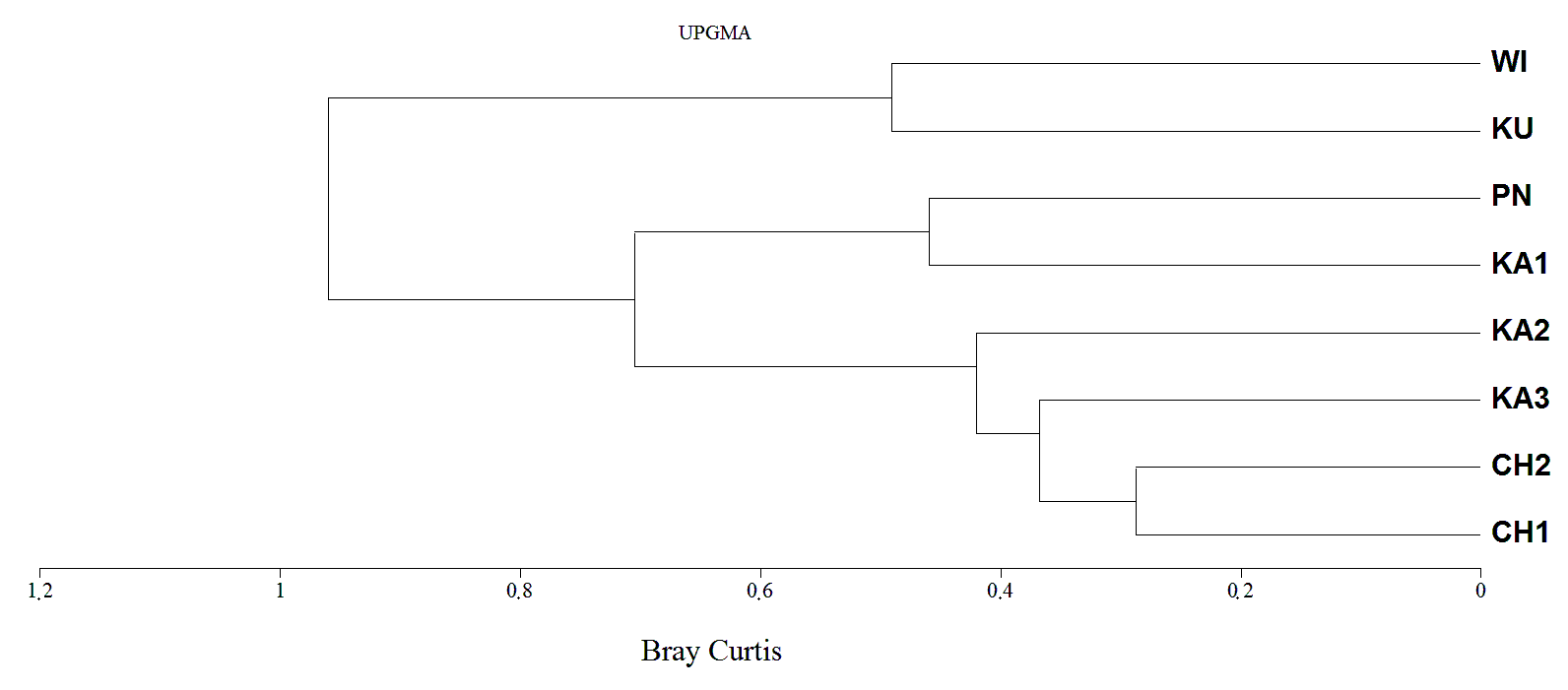
**

**Figure. S3** Dendrogram of faunal similarities between zooplankton communities in analyzed reservoirs.

**
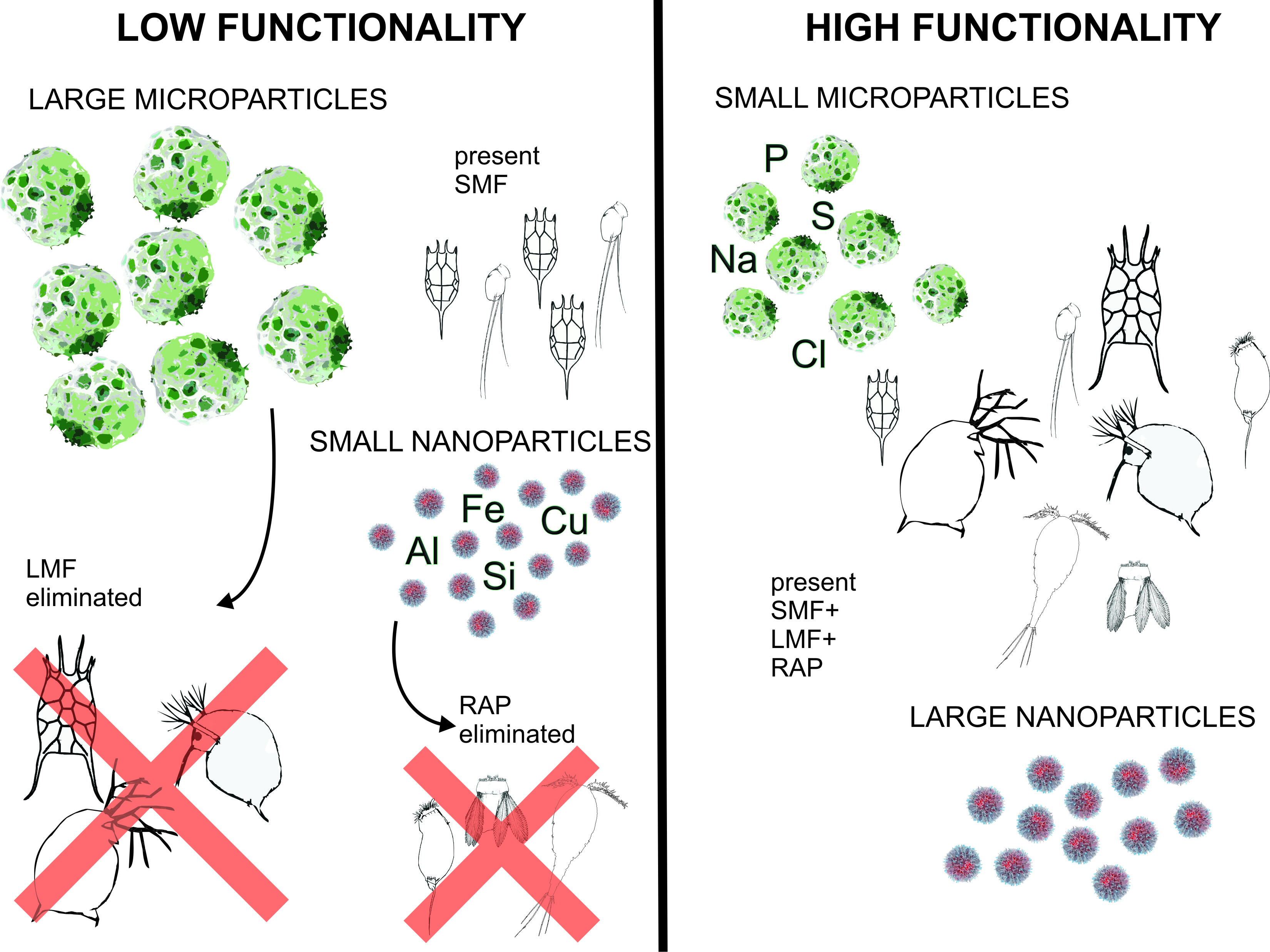
**

**Figure S4.** Diagram of the impact of suspended micro- and nanoparticles on zooplankton functional diversity.
